# Supplementary material for: An Explainable Deep Learning Classifier of Bovine Mastitis Based on Whole-Genome Sequence Data—Circumventing the p >> n Problem
Source: Int J Mol Sci. 2024 Apr 26;25(9):4715. doi: 10.3390/ijms25094715 (PMC11083318; doi:10.3390/ijms25094715)
Supplement: Supplementary file 1 [file ijms-25-04715-s001.zip › Supplementary1.pdf]

C	Threshold	Train LASSO AUC	Test LASSO AUC	Train AUC	Test AUC	Train ACC (Default cut-off value)	Train ACC (Optimal cut-off value)	Test ACC (Default cut-off value)	Test ACC (Optimal cut-off value)
0.1	0.612	1.000	0.420	0.952	0.700	0.742	0.900	0.600	0.650
0.2	0.893	1.000	0.410	0.983	0.505	0.516	0.936	0.500	0.450
0.3	0.745	1.000	0.440	0.956	0.555	0.516	0.936	0.500	0.450
**0.4**	**0.643**	**1.000**	**0.460**	**0.927**	**0.750**	**0.516**	**0.839**	**0.500**	**0.650**
0.5	0.669	1.000	0.480	0.890	0.625	0.677	0.871	0.600	0.550
0.6	0.721	1.000	0.480	0.904	0.400	0.516	0.903	0.500	0.500
0.7	0.550	1.000	0.490	0.908	0.580	0.710	0.903	0.450	0.650
0.8	0.142	1.000	0.480	0.915	0.425	0.484	0.903	0.500	0.300
0.9	0.685	1.000	0.490	1.000	0.700	1.000	1.000	0.700	0.550
1.0	0.685	1.000	0.470	0.908	0.500	0.613	0.839	0.500	0.450

C	Train Sensitivity	Train Specificity	Test Sensitivity	Test Specificity	Train MCC	Test MCC
0.1	1.000	0.800	0.800	0.500	0.747	0.314
0.2	0.875	1.000	0.100	0.800	0.879	-0.140
0.3	1.000	0.867	0.200	0.700	0.878	-0.115
**0.4**	**0.938**	**0.733**	**0.600**	**0.700**	**0.688**	**0.302**
0.5	0.875	0.867	0.600	0.500	0.742	0.101
0.6	0.938	0.867	0.500	0.500	0.742	0.000
0.7	0.875	0.933	0.500	0.800	0.619	0.314
0.8	0.875	0.933	0.200	0.400	0.808	-0.408
0.9	1.000	1.000	0.200	0.900	0.938	0.140
1.0	0.750	0.933	0.300	0.600	0.692	-0.105

Supplementary Table S1. Comparison of performance metrics for various sodels with different C parameters and thresholds. The chosen model in downstream analysis is bolded.
