# Supplementary material for: An Explainable Deep Learning Classifier of Bovine Mastitis Based on Whole-Genome Sequence Data—Circumventing the p >> n Problem
Source: Int J Mol Sci. 2024 Apr 26;25(9):4715. doi: 10.3390/ijms25094715 (PMC11083318; doi:10.3390/ijms25094715)
Supplement: Supplementary file 1 [file ijms-25-04715-s001.zip › Supplementary2.pdf]

GO ID	*Gene Names*	*Number of SNPs*	*FDR_{GO}*
Biological process			
GO:0050804	APP, CLSTN2, CPLX1, DGKI, GRIA1, GRID1, GRID2, GRIK2, GRIK3, GRM8, KIF5B, LRFN2, NEURL1, NRG3, NTRK2, PACSIN2, PCDH17, PLCB4, PLCL1, PLCL2, PRKCB, PRKN, PXK, RASGRF2, SCGN, SHANK1, SLC24A2, SLC8A3, STX3, SYT1, UNC13C	45	3.570E-03
Molecular function			
GO:0005509	ALOX15B, CCBE1, CDH12, CDH5, CDH9, CHP2, CLSTN2, CRACR2B, EDIL3, EHD4, F7, FAT3, FBLN5, FSTL5, GUCA1A, HMCN1, HPCAL1, HSPG2, IQGAP1, KCNIP4, LRP2, NOTCH4, PAM, PCDH15, PCDH17, PCDH7, PLA2G4A, PLA2G5, PLCB4, RYR2, S100A9, SCGN, SCUBE1, SHH, SLIT1, STIM1, SVEP1, SYT1, TGM2, TLL1, TRPM2, UNC13A, UNC13C	70	6.303E-04
GO:0030554	ABCA6, ABCD3, ACOT12, ALPK1, ASCC3, ATP11A, BMPR2, CAMK1D, CAMK2B, CDC42BPB, CDK14, CFTR, CHD7, CLPX, DAPK1, DARS1, DDR2, DGKI, DNAH5, DPH6, EHD4, ENTPD6, EPHA3, EPHA7, HIPK2, HK2, KIF16B, KIF5B, KIT, LONP2, LRRK1, MAPK10, MARK3, MCM6, MLH3, MYH14, MYO18B, MYO1E, MYO3B, MYO5C, MYO6, MYO7A, NADSYN1, NAV3, NEK7, NTRK2, NTRK3, NUBPL, PAK1, PCCA, PDE10A, PDE4A, PDE4B, PFKP, PIK3C2G, PRKACB, PRKAG2, PRKAR2A, PRKCA, PRKCB, PRKCH, PTK2, PXK, RAD51B, RAPGEF4, ROR1, RPS6KA1, RUNX1, SMC1A, STK3, STK33, TDRD9, TPK1, UBE2E2, UBE2E3, UBE2U, ULK1, ZRANB3	141	1.130E-03
GO:0032559	ABCA6, ABCD3, ACOT12, ALPK1, ASCC3, ATP11A, BMPR2, CAMK1D, CAMK2B, CDC42BPB, CDK14, CFTR, CHD7, CLPX, DAPK1, DARS1, DDR2, DGKI, DNAH5, DPH6, EHD4, ENTPD6, EPHA3, EPHA7, HIPK2, HK2, KIF16B, KIF5B, KIT, LONP2, LRRK1, MAPK10, MARK3, MCM6, MLH3, MYH14, MYO18B, MYO1E, MYO3B, MYO5C, MYO6, MYO7A, NADSYN1, NAV3, NEK7, NTRK2, NTRK3, NUBPL, PAK1, PCCA, PDE10A, PDE4A, PDE4B, PFKP, PIK3C2G, PRKACB, PRKAG2, PRKAR2A, PRKCA, PRKCB, PRKCH, PTK2, PXK, RAD51B, RAPGEF4, ROR1, RPS6KA1, RUNX1, SMC1A, STK3, STK33, TDRD9, TPK1, UBE2E2, UBE2E3, UBE2U, ULK1, ZRANB3	141	1.130E-03
GO:0005524	ABCA6, ABCD3, ACOT12, ALPK1, ASCC3, ATP11A, BMPR2, CAMK1D, CAMK2B, CDC42BPB, CDK14, CFTR, CHD7, CLPX, DAPK1, DARS1, DDR2, DGKI, DNAH5, DPH6, EHD4, ENTPD6, EPHA3, EPHA7, HIPK2, HK2, KIF16B, KIF5B, KIT, LONP2, LRRK1, MAPK10, MARK3, MCM6, MLH3, MYH14, MYO18B, MYO1E, MYO3B, MYO5C, MYO6, MYO7A, NADSYN1, NAV3, NEK7, NTRK2, NTRK3, NUBPL, PAK1, PCCA, PFKP, PIK3C2G, PRKACB, PRKAG2, PRKCA, PRKCB, PRKCH, PTK2, PXK, RAD51B, ROR1, RPS6KA1, RUNX1, SMC1A, STK3, STK33, TDRD9, TPK1, UBE2E2, UBE2E3, UBE2U, ULK1, ZRANB3	136	4.745E-03
GO:0032553	ABCA6, ABCD3, ACOT12, ALPK1, ARF4, ASCC3, ATP11A, BMPR2, CAMK1D, CAMK2B, CDC42BPB, CDK14, CFTR, CHD7, CLPX, CNGB3, DAPK1, DARS1, DDR2, DGKI, DNAH5, DPH6, EHD4, ENTPD6, EPHA3, EPHA7, GTPBP10, GUCY1A2, HIPK2, HK2, KIF16B, KIF5B, KIT, LONP2, LRRK1, MAPK10, MARK3, MCM6, MFN1, MLH3, MYH14, MYO18B, MYO1E, MYO3B, MYO5C, MYO6, MYO7A, NADSYN1, NAV3, NEK7, NOS1, NTRK2, NTRK3, NUBPL, PAK1, PCCA, PDE10A, PDE4A, PDE4B, PFKP, PIK3C2G, PRKACB, PRKAG2, PRKAR2A, PRKCA, PRKCB, PRKCH, PTK2, PXK, RAB30, RAB7B, RAD51B, RAPGEF4, RHOBTB3, ROR1, RPS6KA1, RRAGD, RUNX1, SEPTIN9, SMC1A, SRP54, STK3, STK33, TDRD9, TGM2, TPK1, UBE2E2, UBE2E3, UBE2U, ULK1, ZRANB3	161	4.745E-03
GO:0032555	ABCA6, ABCD3, ACOT12, ALPK1, ARF4, ASCC3, ATP11A, BMPR2, CAMK1D, CAMK2B, CDC42BPB, CDK14, CFTR, CHD7, CLPX, CNGB3, DAPK1, DARS1, DDR2, DGKI, DNAH5, DPH6, EHD4, ENTPD6, EPHA3, EPHA7, GTPBP10, GUCY1A2, HIPK2, HK2, KIF16B, KIF5B, KIT, LONP2, LRRK1, MAPK10, MARK3, MCM6, MFN1, MLH3, MYH14, MYO18B, MYO1E, MYO3B, MYO5C, MYO6, MYO7A, NADSYN1, NAV3, NEK7, NTRK2, NTRK3, NUBPL, PAK1, PCCA, PDE10A, PDE4A, PDE4B, PFKP, PIK3C2G, PRKACB, PRKAG2, PRKAR2A, PRKCA, PRKCB, PRKCH, PTK2, PXK, RAB30, RAB7B, RAD51B, RAPGEF4, RHOBTB3, ROR1, RPS6KA1, RRAGD, RUNX1, SEPTIN9, SMC1A, SRP54, STK3, STK33, TDRD9, TGM2, TPK1, UBE2E2, UBE2E3, UBE2U, ULK1, ZRANB3	160	4.756E-03
GO:0017076	ABCA6, ABCD3, ACOT12, ALPK1, ARF4, ASCC3, ATP11A, BMPR2, CAMK1D, CAMK2B, CDC42BPB, CDK14, CFTR, CHD7, CLPX, CNGB3, DAPK1, DARS1, DDR2, DGKI, DNAH5, DPH6, EHD4, ENTPD6, EPHA3, EPHA7, GTPBP10, GUCY1A2, HIPK2, HK2, KIF16B, KIF5B, KIT, LONP2, LRRK1, MAPK10, MARK3, MCM6, MFN1, MLH3, MYH14, MYO18B, MYO1E, MYO3B, MYO5C, MYO6, MYO7A, NADSYN1, NAV3, NEK7, NTRK2, NTRK3, NUBPL, PAK1, PCCA, PDE10A, PDE4A, PDE4B, PFKP, PIK3C2G, PRKACB, PRKAG2, PRKAR2A, PRKCA, PRKCB, PRKCH, PTK2, PXK, RAB30, RAB7B, RAD51B, RAPGEF4, RHOBTB3, ROR1, RPS6KA1,	160	5.599E-03

	RRAGD, RUNX1, SEPTIN9, SMC1A, SRP54, STK3, STK33, TDRD9, TGM2, TPK1, UBE2E2, UBE2E3, UBE2U, ULK1, ZRANB3		
GO:0005216	ANO10, ANO5, CACNA1D, CACNA1S, CACNA2D3, CFTR, CNGB3, GABRB1, GRIA1, GRID1, GRID2, GRIK2, GRIK3, KCNC3, KCNC4, KCNH1, KCNIP4, KCNK2, KCNK9, LRRC52, PIEZO2, RYR2, SEC61A1, SLC24A2, SLC24A4, SLC26A7, SLC9C1, TMEM63B, TRPC6, TRPM2	39	4.511E-02
GO:0031267	AKAP13, CAV1, CYFIP2, DGKI, DOCK4, IQGAP1, IQGAP2, PAK1, PLCE1, PRKCH, RAB11FIP2, RAB3GAP2, RAPGEF4, RASSF5, RHOBTB3, SYTL3, ULK1, WASF1, YIPF2	26	4.511E-02
GO:0005096	ABR, ARHGAP10, ARHGAP22, ARHGAP29, ARHGAP31, ARHGAP36, DAB2IP, ELMOD3, IQGAP1, IQGAP2, RAB3GAP2, RALGAPA2, RAP1GDS1, RASA3, SRGAP1, TBC1D17, TBC1D4, TBC1D5	24	4.786E-02
GO:0017075	CPLX1, DAPK1, SYBU, SYT1, UNC13A, UNC13C	7	4.786E-02
GO:0022838	ANO10, ANO5, CACNA1D, CACNA1S, CACNA2D3, CFTR, CNGB3, GABRB1, GRIA1, GRID1, GRID2, GRIK2, GRIK3, KCNC3, KCNC4, KCNH1, KCNIP4, KCNK2, KCNK9, LRRC52, PIEZO2, RYR2, SEC61A1, SLC24A2, SLC24A4, SLC26A7, SLC9C1, TMEM63B, TRPC6, TRPM2	39	4.786E-02
GO:0008276	ASH1L, ATPSCKMT, CARM1, LCMT1, NSD1, PCMTD1, SETDB2, SMYD3, SUV39H1, WDR5	12	4.890E-02

Supplementary Table S2. Significant GO terms with related gene names and SNP numbers inside them. The enrichment analysis was carried out on significant SNPs subset based on selected DL-based SNPs set. The GO terms overlapping between the GWAS-based and the DL-based significant enrichment have been underlined.
